# Comparison of walking performance over the first 2 minutes and the full 6 minutes of the Six-Minute Walk Test

**DOI:** 10.1186/1756-0500-7-269

**Published:** 2014-04-25

**Authors:** Richard W Bohannon, Deborah Bubela, Susan Magasi, Heather McCreath, Ying-Chih Wang, David Reuben, William Z Rymer, Richard Gershon

**Affiliations:** 1 Physical Therapy Program, Department of Kinesiology, Neag School of Education, University of Connecticut, Storrs, CT 06269-2101, USA; 2 Department of Medical Social Sciences, Feinberg School of Medicine, Northwestern University, Chicago, IL 60611, USA; 3 Division of Geriatrics, David Geffen School of Medicine, University of California Los Angeles, Los Angeles, CA 90095, USA; 4 Department of Occupational Science & Technology, University of Wisconsin-Milwaukee, Milwaukee, WI 53201, USA; 5 Sensory Motor Performance Program, Rehabilitation Institute of Chicago, Northwestern University, Chicago, IL 60611, USA; 6 University of Illinois, Chicago, USA

## Abstract

**Background:**

Although the Six-Minute Walk Test (6MWT), as recommended by the American Thoracic Society, is widely used as a measure of functional endurance, it may not be applicable in some settings and populations. We sought to examine, therefore, performance over the first 2 minutes and the full 6 minutes of the 6MWT. Specifically, we investigated completion rates, distances walked, test-retest reliability, and the relationship between distances walked over the first 2 and the full 6 minutes of the 6MWT.

**Methods:**

Community-dwelling children and adults age 3–85 years (n = 337) were asked to walk back and forth on a 15.24 meter (50 ft) course as far as possible without running over a 6 minute period. Test completion and the distance covered by the participants at 2 and 6 minutes were documented. The reliability of distances covered at 2 and 6 minutes was determined by retesting a subsample of 54 participants 6 to 10 days later. The relationship between distances covered at 2 and 6 minutes was determined for the 330 participants completing the 6MWT.

**Results:**

All 337 participants completed at least 2 minutes of walking, but 7 children less than 5 years of age ceased walking before 6 minutes had elapsed. For the remaining 330 participants the mean distance walked was 186 meters at 2 minutes and 543 meters at 6 minutes. The distances covered at 2 and 6 minutes were reliable between sessions (intraclass correlation coefficients = 0.888 and 0.917, respectively). The distances covered over 2 and 6 minutes were highly correlated (r = 0.968).

**Conclusions:**

The completion rate, values obtained, test-retest reliability, and relationship of the distances walked in 2 and 6 minutes support documentation of 2 minute distance during the 6MWT. The findings also provide support for use of a Two-Minute Walk Test as the endurance component in the Motor Battery of the NIH Toolbox.

## Background

Functional endurance is necessary for individuals to live independently without accommodation in community settings [1]. Walk tests, in which the distance covered over a period of time is documented, have been used since at least the 1970s to quantify functional endurance [2]. Though most widely used among patients with pulmonary [2-4] or cardiac [5,6] diagnoses, walk tests have also been employed for patients with neurologic problems [7,8], amputations [9], circulatory insufficiency [10], orthopedic conditions [11] and renal [12] or liver [13] disease. The tests have been utilized with community-dwelling children [14,15] and adults [16,17] as well. Walk tests described in the literature range in duration from 1 to 12 minutes [2,3,8], but the Six-Minute Walk Test (6MWT) is probably the most frequently used. The American Thoracic Society has recommended the 6MWT and published guidelines for its administration [18].

Broad use and the American Thoracic Society’s recommendation notwithstanding, the duration of the 6MWT renders its use impracticable in busy settings- particularly if numerous individuals need to be tested over a limited time span. Moreover, some individuals are unable or unwilling to complete the 6MWT, even with allowable standing rests [19-21], resulting in null values. This fact has led to the use and recommendation of shorter duration walk tests- most notably the Two-Minute Walk Test (2MWT) [19,20,22]. To date, the completion of 2 minutes of walking and the distance walked over 2 minutes has not been documented over the 3 to 85 year age span. Good intra-rater and interrater reliability have been described for distances achieved during 2MWTs, at least for older adults residing in long-term care [20] and patients with stroke [7]. However, the reliability of distances covered during the first 2 minutes of the 6MWT has not been described. Distances traversed during a 2MWT and 6MWT have been shown to be highly correlated (r = 0.930-0.997) [4,7,20,23], but the relationship between the distance walked during the first 2 minutes of the 6MWT and the distance covered over the full course of the 6MWT is not established. If the completion rate for 2 minutes of walking surpasses that of 6 minutes of walking and if the distance covered during the first 2 minutes of the 6MWT is reliable and strongly related to the 6MWT distance, measurement of the distance covered in the first 2 minutes might be justified and perhaps considered as a substitute for the 6MWT distance. The purpose of this study, therefore, was to describe completion rates and distance walked, determine the test-retest reliability for the distances walked, and ascertain the relationship between the distance walked in the first 2 minutes and full 6 minutes of the 6MWT.

## Methods

This investigation was part of the validation phase of the Motor Domain of the National Institutes of Health (NIH) Toolbox for the Assessment of Neurological and Behavioral Function (NIH Toolbox) study [24]. It included data gathered from convenience samples tested at 3 participating sites (University of Connecticut, Rehabilitation Institute of Chicago, and University of California Los Angeles). The institutional review boards from each site approved the study. All participants or their legal guardians provided written informed consent before testing. Participants were 3–85 years of age. Inclusion required that participants were fluent in English, were able to walk without use of an assistive device, and had no heart, vascular, lung, or bone/joint problems that precluded their ability to walk independently.

Prior to obtaining candidate measures for the NIH Toolbox, basic demographic (age, gender), anthropometric (height, weight [body mass index]), and health status information was obtained along with baseline heart-rate and blood pressure measurements. Thereafter, in random order, participants completed the 6MWT as either the first or last task of the larger battery of tests. Participants were asked to walk as far as possible without running for 6 minutes. They walked on a flat, hard and straight 15.24 meter (50 foot) course marked on both ends with traffic cones. At 1 minute intervals they were told that they were “doing well” or to “keep up the good work” and informed of the time remaining. Participants were allowed to take a standing rest if they chose to do so at any point, but were asked to resume walking if and when possible until the 6 minute period had elapsed. The distances covered in 2 and 6 minutes were documented. A subset of 54 participants distributed across age and sex strata repeated the walk tests within a 6–10 day interval to examine test-retest reliability of the measures. These participants were the first who agreed to return for retesting.

Performance over the course of the 6MWT and across the age-span was first described by noting the number of participants not completing the 6MWT. For individuals completing the 6MWT descriptive statistics (range, mean, standard deviation, and confidence intervals) were calculated for the distances walked in 2 and 6 minutes. Intraclass correlation coefficients (ICCs) were used to describe the reliability of the distances walked in 2 and 6 minutes. A Pearson correlation and linear regression were used to determine the relationship between the distances covered over the first 2 and the total 6 minutes of the 6MWT. Statistical analyses were completed using SPSS 15.0 with an alpha level of 0.05.

## Results

Table 1 summarizes demographic and other nonperformance data describing the 330 participants who completed the 6MWT. Approximately 55% of the participants were female and about 35% identified their race as other than White. Most participants reported their health to be excellent (49%) or very good (36%).

**Table 1:** Number (%) of participants completing walk task by demographic characteristics

**Variable**	**N (%)**
Site*	
University of Connecticut	137 (41.5)
Rehabilitation Institute of Chicago	116 (35.2)
University of California, Los Angeles	77 (23.3)
Total	330 (100)
Gender	
Female	180 (54.5)
Male	150 (45.5)
Age (yrs) strata per study design*	
3-6	58 (17.6)
7-13	78 (23.6)
14-20	33 (10.0)
21-65	100 (30.3)
66-85	61 (18.5)
Hispanic or Latino	37 (11.2)
Race	
White	211 (63.9)
Asian	50 (15.2)
Black	29 (8.8)
Other	40 (12.1)

*Due to research design features, UCLA enrolled only children.

Seven of 337 individuals (2.1%) who enrolled in the study did not complete the 6MWT. All 7 were less than 5 years old. One (0.3%) of the 7 refused to attempt the task. The other 6 (1.8%) walked at least 2 minutes but stopped before 6-minutes had elapsed and declined to resume walking for nonphysical reasons (e.g., averted attention).

**Figure 1:**
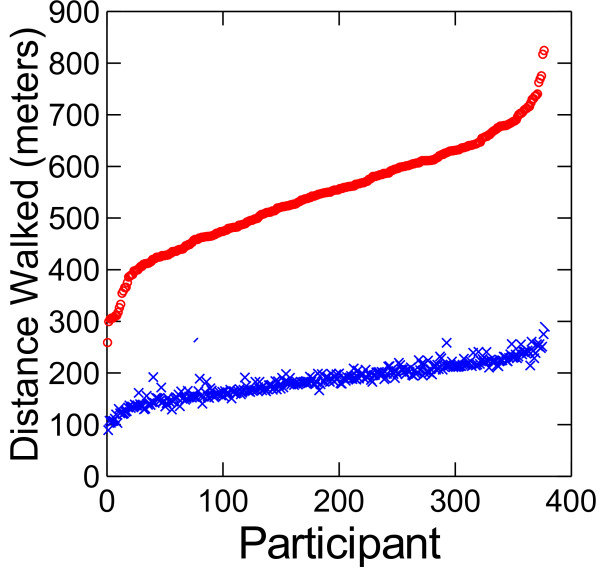
Distance walked by individual participants over 6 minutes (red) and 2 minutes (blue) of the Six-Minute Walk Test.

**Figure 2:**
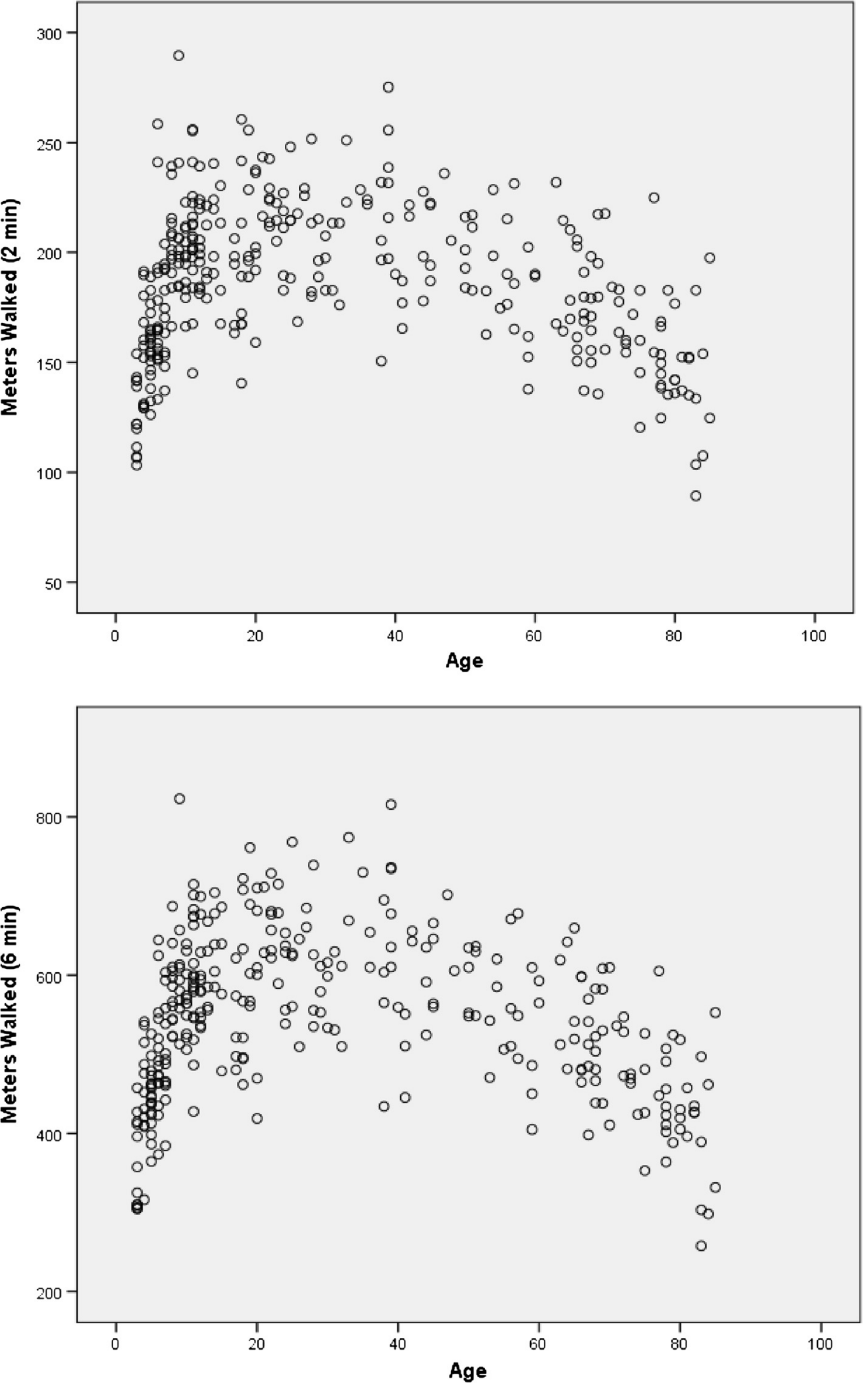
Scatterplots showing the relationship between age and distance walked in 2 minutes (top) and 6 minutes (bottom) of the Six-Minute Walk Test.

For the 330 individuals completing the 6MWT, only 1 required a standing rest stop. The distance walked over the course of 2 minutes was 186 ± 34 (91–290) meters. Over the full 6 minutes the distance walked was 543 ± 102 (258–823) meters. Individual performance varied considerably (Figure 1). Only participants aged less than 7 years or more than 74 years walked 135 meters or less in 2 minutes (n = 23) or 396 meters or less in 6 minutes (n = 22) (Figure 2). Excellent test-retest reliability was demonstrated for the distances covered over the first 2 minutes (ICC = 0.888 [0.814- = 0.933]) and the full 6 minutes (ICC = 0.917 [0.862-0.951]) of the 6MWT. The distances covered over 2 and 6 minutes were correlated highly (r = 0.968, p < 0.0001). The linear regression equation describing the relationship was: 6MWT distance (meters) = 27 meters + 2.87*2 minute distance (meters).

## Discussion

Timed walk tests are commonly used to measure the functional endurance of diverse populations [1-23]. Among such tests, the 6MWT, as recommended by the American Thoracic Society, is probably the most widely used. This led to its inclusion in the validation phase of the NIH Toolbox project [24]. In the validation phase we were particularly interested in the rate of test completion, test performance, test-retest reliability, and the degree to which the distances walked over earlier minutes of the test were reflective of the distance covered over the full 6-minute duration of the 6MWT.

The completion rate we observed for the 6MWT was 97.9%. Only individuals less than 5 years old failed to complete the test. The completion rate among the children we tested was less than the 100% reported by Lammers et al. for 4–11 year old children [14] and greater than the 82.5% reported by Geiger et al. for boys and girls 3–18 years of age [21]. Among a subset of children 3–5 years of age in their study, Geiger et al. reported a “response rate” of only 39%, though they did not operationalize “response rate”. All of the community-dwelling older adults we studied completed the 6MWT. Our sample, therefore, was more able than that of Brooks et al. [19]. Of their older adults, who were undergoing rehabilitation, only 1 of 8 was able to walk for 6 minutes. As a consequence they abandoned further testing with the 6MWT and resorted to using a 2MWT. Fifty of 52 older adults tested by Brooks et al. were able to complete a 2MWT. Gijbels et al., who tested patients with multiple sclerosis, have suggested that “the last 4-minute period of the 6MWT seems redundant” [23]. Together these findings support the measurement of distance walked over 2 minutes, whether it be the first 2 minutes of a 6MWT or a 2MWT. By capturing the distance walked over 2 minutes null values might be avoided in clinical documentation and research trials.

We documented the distances walked by our convenience sample of participants over 6 minutes and over the first 2 minutes of the 6MWT. We used a back-and-forth distance of 15.2 meters rather than the 30.5 meter distance recommended by the American Thoracic Society [18] as we judged it to be more ecologically generalizable. Although a greater frequency of turns has the potential to suppress the distance walked over time [25], the distances we report for 6 minutes appear to be relatively comparable to those reported by others testing able-bodied children and adults [14,26-29]. Specifically, the mean distance walked by children (3–11 years) in our study was 510 meters compared to 470 ± 59 meters in the study of Lammers et al. [14]. The median distance covered by adults (40 or more years) in our study was 512 meters compared to 527 meters in the study of Enright and Sherrill [29]. Regarding the distance walked by our participants in the first 2 minutes of the 6MWT (186 meters), it far exceeded mean 2MWT distances for older adults in long-term care [20], patients with lower-limb amputations [9], patients undergoing cardiac surgery [22], and patients with stroke [7]. Providing additional perspective, the mean distance our participants walked in the first 2 minutes of the 6MWT also exceeded the mean distances required to traverse a post office (52.0 meters), bank (57.1 meters), or medical office building (65.8 meters); the mean distance covered over the full 6 minutes exceeded the mean distances needed to traverse a pharmacy (206.3 meters), department store (345.9 meters), or grocery store (380.6 meters) [30]. So even if we do not consider our 2-minute or 6-minute distances to be normative, they are comparable to data obtained from other community-dwelling individuals, greater than data obtained from patient groups, and ecologically relevant.

By measuring performance at 2 and 6 minutes of the 6MWT on 2 occasions, we were able to examine the reliability of distances covered at both times. While the ICC for 6 minutes was of greater magnitude than the ICC for 2 minutes, both are indicative of good reliability and fall within the confidence intervals of one another. Moreover, the ICCs for 2 minutes are within the range of those reported for patients with stroke (ICC = 0.85) [7] and for older adults in long-term care (0.94 and 0.95) [20].

Although we did not separately examine participants using a 2MWT and 6MWT, the correlation between the distances walked at 2 and 6 minutes of the 6MWT (0.968) is similar to correlations reported by researchers comparing 2MWT and 6MWT performance among older adults in long-term care (0.930) [20], and patients with multiple sclerosis (0.980) [23], stroke (0.997) [7], and chronic obstructive pulmonary disease (0.937) [4]. Together, these findings demonstrate that the distance walked in 2 minutes is a valid indicator of the distance covered in 6 minutes, whether the 2 minute distance is obtained from a 6MWT or a separate 2MWT.

This study had several limitations. First a convenience sample of relatively healthy community-dwelling individuals was used. A population-based sample might yield different completion rates, reliability coefficients, and relationships between distances walked. Patients with limited aerobic capacity or pain might also demonstrate lower completion rates. Depending on the frequency, timing, and duration of standing rests, reliability and correlations between distances walked at 2 and 6 minutes could be depressed relative to those we found. Our study provides no indication of the relative discriminant or predictive validity of the distance walked in 2 versus 6 minutes of the 6MWT. Second, we only examined 2-minute walk distance in the context of a 6MWT. The comparability of distance walked in a 2MWT in our sample is therefore unknown. Individuals may walk faster if they realize they won’t have to walk another 4 minutes. Third, though our sample was relatively large, we considered it to be too small to perform subgroup analysis on specific strata (e.g., girls 3–5 years). Finally, we did not measure oxygen consumption, heart rate or perceived exertion over the course of the 6MWT. While doing so is beyond the scope of the NIH Toolbox project, such measures can be informative in as to specifics over the course of 6 minutes. Motl et al., for example, measured oxygen consumption at 30 second intervals of the 6MWT and found that steady-state aerobic metabolism was not reached until 3 minutes of walking were completed in patients with multiple sclerosis [31].

Future research will involve the collection of normative data for the 2MWT across the age-span. That data will then be used as a benchmark for various community-dwelling and patient groups.

## Conclusions

Based on completion rates, distances walked, reliability and the high correlation between the distance walked in 2 and 6 minutes, the distance walked over 2 minutes can be considered to be a legitimate alternative to the distance walked over 6 minutes for indicating functional endurance among relatively healthy community-dwelling individuals. Even if the 6MWT is used, it may be useful to document 2-minute walk distance so that useful information is still obtained from individuals unable to complete the full 6MWT. The 2MWT will be used as the measure of functional endurance in the norming phase of the NIH Toolbox project.

## Competing interests

None of the authors have any competing financial or non-financial competing interests relating to the manuscript or the content of this manuscript.

## Authors’ contributions

RB and DB collected and analyzed data and drafted the manuscript. SM, HM, and YW developed protocol, collected data and contributed input to the manuscript. DR, WZR, and RG conceived of the large scale NIH Toolbox study and participated in the design and coordination of the research efforts. All authors read and approved the final manuscript.
